# A Survey-Based Study Examining Differences in Perception of Postural Orthostatic Tachycardia Syndrome Between Patients and Primary Care Physicians

**DOI:** 10.7759/cureus.30167

**Published:** 2022-10-11

**Authors:** Jessica Cooperrider, Jennifer Kriegler, Samina Yunus, Robert Wilson

**Affiliations:** 1 Neurology, Cleveland Clinic Foundation, Cleveland, USA; 2 Family Medicine, Cleveland Clinic Foundation, Cleveland, USA

**Keywords:** dizziness, migraine headaches, hyperadrenergic pots, near syncope, syncope, clinically significant fatigue, fatigue, migraine disorder, neuropathic pots, pots

## Abstract

Introduction

Postural orthostatic tachycardia syndrome (POTS) is an underdiagnosed and undertreated dysautonomia. We hypothesize that there are differences between primary care physicians (PCPs) and patients’ perceptions of POTS and that correcting these discrepancies may improve patient care.

Methods

Two groups were surveyed: Patients who received care for POTS symptoms from a Cleveland Clinic neurologist or nurse practitioner and patients who received care from Cleveland Clinic family medicine or internal medicine physicians.

Results

PCPs (81%) rated lightheadedness as the symptom with the most significant negative impact on patient's quality of life with POTS, while patients rated fatigue (32%) as their worst symptom. PCPs were somewhat comfortable managing POTS but were less confident recommending cardiac rehabilitation and daily behavioral measures.

Conclusion

PCPs may need to continue review the negative impact of fatigue on the quality of life of POTS patients as well symptoms of body pain and lightheadedness. Although they are relatively comfortable managing POTS, PCPs may benefit from training on several aspects of POTS treatment.

## Introduction

Postural orthostatic tachycardia syndrome (POTS) is an autonomic syndrome that is characterized by symptoms including lightheadedness, fatigue, palpitations, and increased upright heart rate with the maintenance of normal blood pressure [1]. Although this diagnosis is thought to affect 500,000 and 3,000,000 Americans, most patients report seeing physicians who have not heard of POTS [2]. The average time until the diagnosis is four years, with patients seeing an average of seven physicians before receiving a POTS diagnosis [2]. Furthermore, wait times to see a POTS specialist range from 6 to 36 months [2]. Patients are also frequently misdiagnosed, with 75% of patients reporting prior misdiagnoses [3]. In addition, there is now a clear link between COVID-19 infection and the development of POTS. As a result, as never before, there is a critical need for providers skilled in identifying and caring for patients with POTS [4].

There is limited literature on the knowledge of primary care physicians (PCPs) regarding POTS. In this study, we surveyed Cleveland Clinic patients with POTS and PCPs in order to determine: 1) The symptoms and healthcare access of patients, 2) The difference in perception of POTS between patients with POTS and PCPs, and 3) Whether PCPs judge themselves as having the knowledge to treat POTS patients. We hypothesize that there is a difference between the symptoms that POTS patients find most disruptive and what physicians think is most disruptive. Furthermore, we hypothesize that PCPs feel relatively undertrained in treating POTS patients. By highlighting gaps in physicians' knowledge, future physicians could seek training to fill these gaps, improving the time to diagnose and manage symptoms for patients with POTS.

## Materials and methods

Participants

Two populations were sampled: 1) Patients who had seen a Cleveland Clinic neurologist or neurology nurse practitioner for management of POTS (April-September 2020), and 2) PCPs at Cleveland Clinic, including both family medicine and internal medicine physicians. Patients were drawn from the neurology clinic because there was the highest likelihood of an accurate diagnosis of POTS. In addition, this research was approved by the Cleveland Clinic IRB as exempt research due to minimal risk.

Survey administration

Surveys were intended to be simple to improve the survey completion rate. For patients, survey questions interrogated diagnosis, symptoms, access to care, emergency department visits, and perception of PCP comfort level with POTS. For PCPs, surveys inquired about the perception of patients' symptoms and comfort level with POTS treatments. Patients were emailed a survey invitation through the patient electronic medical record portal at Cleveland Clinic, MyChart. This message described the study and contained a link to an anonymous REsearch Data CAPture (REDCap) survey (Appendix 1). A follow-up MyChart message was sent one week later asking if patients would consider completing the survey again. PCPs were invited to participate through an email containing a study description and a link to an anonymous REDCap survey (Appendix 2).

Data analysis

Survey results were stored in the REDCap online database. If individuals had completed the survey twice, the latter survey was excluded from the analysis. Data are presented as a percentage of total or mean ± SD. Comparisons between collected variables were calculated with a t-test or chi-square test. Significance was set at p<0.05.

## Results

A total of 479 patients were invited to participate in this study. Two hundred twenty-six surveys were collected, but after the removal of duplicate surveys, n=190 surveys remained for analysis. A total of 280 PCPs were invited to participate in this study, and n=80 surveys were completed and analyzed.

Patient results

Diagnosis and Symptoms

**Figure 1:**
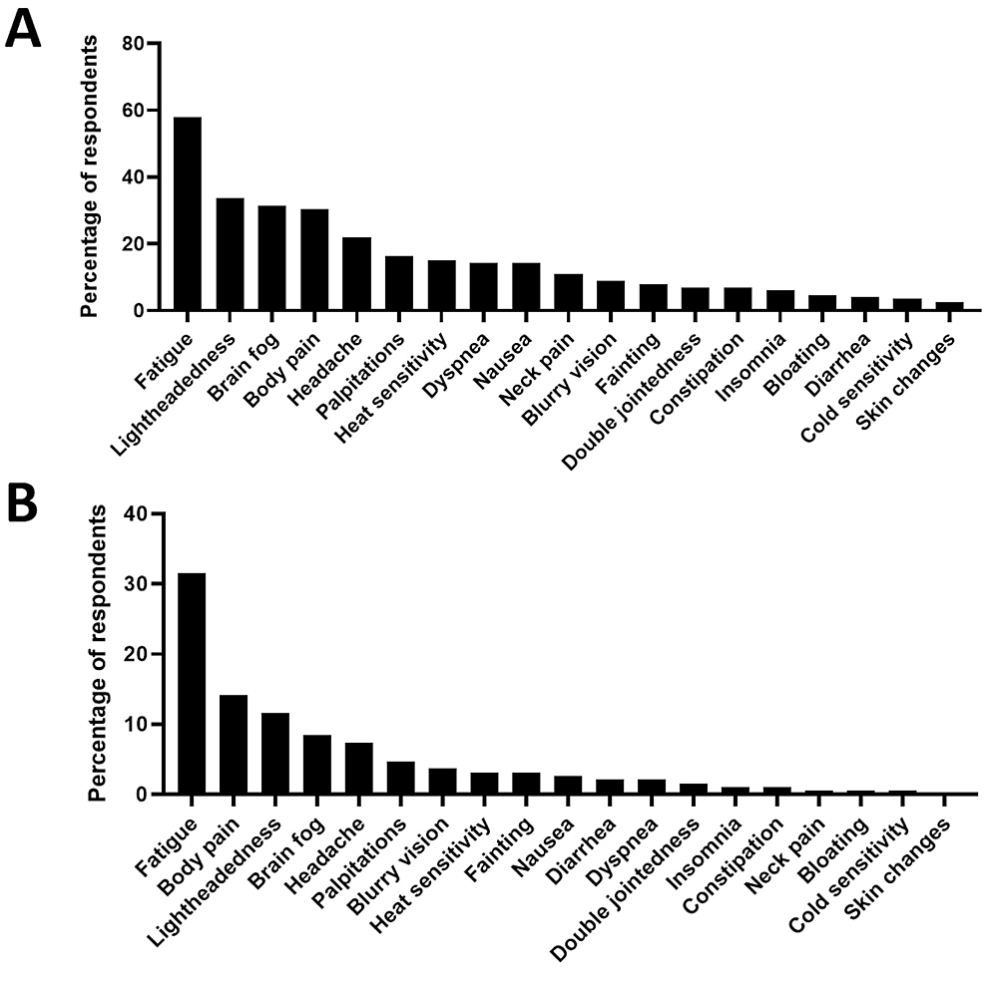
A) Most common occurring symptom, B) Symptom with greatest impact on quality of life.

A total of 81% of patients reported being diagnosed with POTS by a neurologist, 16% by a cardiologist, 2% by PCP, and the remaining 1% reported "other." The most commonly occurring symptom was fatigue (58%), followed by lightheadedness (34%), brain fog (32%), body pain (31%), headache (22%), and other symptoms (Figure 1A). The symptom that had the most significant impact on quality of life was fatigue (32%), followed by body pain (14%), lightheadedness (12%), brain fog (8%), headache (7%), and other symptoms (Figure 1B).

Treatment and clinical experience

A total of 87% of patients reported that their POTS symptoms were treated by a neurologist, 4% by a cardiologist, 7% reported no one was treating their symptoms, and the remaining patients reported that multiple or other physicians treated their symptoms.

A total of 78% of patients reported that they had been seen by a physician who was not familiar with POTS. In addition, 42% of patients reported seeing a physician who manages POTS symptoms three or more times per year, 29% saw a physician who manages POTS symptoms twice a year, 18% once a year, and 11% not at all in a year. Patients rated the ease at which they could obtain an appointment with their POTS physician as 4.5 ± 2.7 (mean ± SD), with 0=very difficult, 5=neutral, and 10=very easy.

Most patients (66%) contacted the physician who treats POTS symptoms multiple times per year via the Cleveland Clinic online patient portal, MyChart. A total of 20% reported contacting their physician multiple times per month, 7% once per year, and 7% never. In addition, most patients (53%) traveled greater than 60 miles to see their POTS physician, while 20% traveled 0-15 miles, 18% traveled 15-30 miles, and 9% traveled 30-60 miles.

Emergency department usage

**Figure 2:**
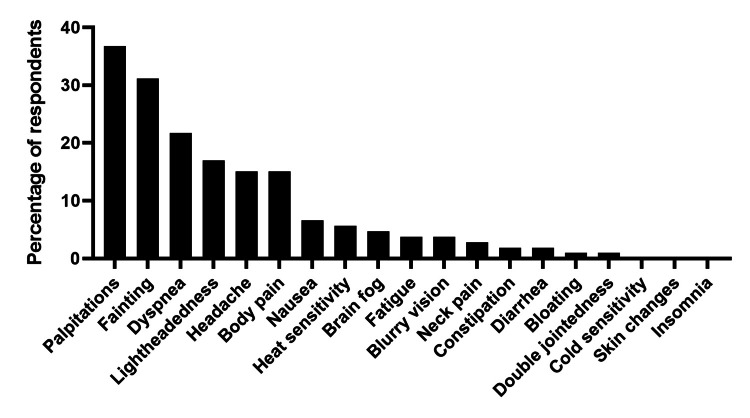
Symptoms that lead to ED visits.

A total of 56% of patients reported visiting the ED for treatment of POTS in the past two years. A total of 27% of patients had visited the ED three or more times, 10% twice, and 19% once. The most reported symptom leading to an ED or urgent care visit was palpitations (37%), followed by fainting (31%) and dyspnea (22%) (Figure 2). Patients who more frequently utilized the ED (defined here as three or more visits in two years) and patients who infrequently utilized the ED (defined as one or zero ED visits in two years), both described their most common POTS symptoms as fatigue (59% of frequent utilizers vs. 57% of infrequent). However, more frequent utilizers were much more likely to faint (22% of frequent utilizers fainted vs. 2% of infrequent utilizers, p<0.0001) and less likely to have headaches (12% of frequent utilizers have headaches vs. 28% of low utilizers, p=0.03).

Patient perception of PCPs

A total of 97% of patients reported having a PCP. Of these patients, 42% thought their PCP was familiar with POTS, 30% thought their PCP was unfamiliar with POTS, and 27% were unsure. In addition, patients thought that their PCP was moderately comfortable managing POTS, rating their comfort level as 4.3 ± 2.6 on a scale from 0-10, with 0=very uncomfortable and 10=very comfortable.

PCP results

Diagnosis and Symptoms

**Figure 3:**
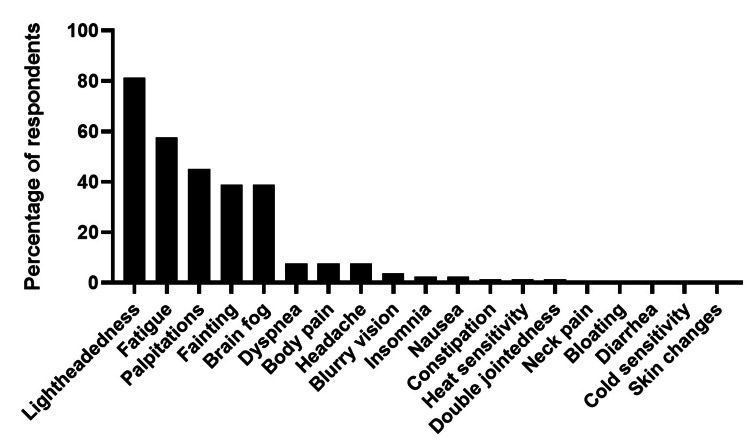
PCPs thoughts about POTS patients' symptoms causing a negative impact on their quality of life. PCP: PRimary care physician; POTS: Postural orthostatic tachycardia syndrome.

Most PCPs in this study (81%) spent most of their clinical time seeing patients at community health centers and not at the Cleveland Clinic's main campus. PCPs were moderately comfortable diagnosing POTS, ranking their comfort level as 4.2 ± 2.9 on a scale from 0 to 10. The symptom that PCPs thought had the greatest negative impact on the quality of life of POTS patients was lightheadedness (81%), followed by fatigue (58%), palpitations (45%), fainting (39%), and brain fog (39%) (Figure 3).

Clinical management

A total of 44% of PCPs saw POTS patients twice a year, 33% saw patients monthly, 21% yearly or less frequently, and 3% weekly. A total of 55% of PCPs surveyed reported treating POTS patients for their POTS symptoms, and 91% of PCPs replied "yes" to the question of whether POTS patients were challenging to manage. However, on a scale of 0-10, PCPs ranked themselves as moderately comfortable managing POTS, ranking their comfort as 5.1 ± 2.6 on a scale from 0-10, significantly different from the patient rating of PCP comfort (5.1 vs. 4.3, respectively, p=0.02). Of those PCPs who treat POTS, 82% prescribe pharmacologic treatment, 66% suggest daily behavioral measures to improve POTS symptoms, and 36% prescribe cardiac rehabilitation. The comfort level with prescribing pharmacologic treatment was 4.1 ± 2.6. Most PCPs were not familiar with cardiac rehabilitation for the treatment of POTS (59%), and 44% were not familiar with daily behavioral measures for the treatment of POTS. PCPs thought that it was somewhat challenging for POTS patients to schedule an appointment with their POTS physician, ranking it 3.8 ± 2.1 out of a scale of 0-10.

## Discussion

The prevalence of POTS is thought to be underestimated, although it is one of the more common disorders of the autonomic nervous system [5]. Furthermore, post-COVID-19 autonomic dysfunction resulting in POTS [6] will almost certainly increase the burden of dysautonomia on the healthcare system. As such, evaluating whether PCPs feel comfortable diagnosing and managing POTS in order to target areas for further education may improve access and quality of care for people with dysautonomia.

In this study, POTS patients most frequently experienced fatigue, lightheadedness, and brain fog, similar to other recent studies [3,7], but palpitations and dyspnea occurred less frequently than in previous studies. Differences in symptom proportions are likely due to the way in which we interrogated symptoms. We asked patients for their three most common symptoms and not to exhaustively affirm whether they experienced a list of symptoms. Interestingly, after fatigue, body pain was the symptom that contributed most to a negative quality of life. The exact type of pain patients are referring to is unclear; future work delving into pain and examining possible comorbidity with fibromyalgia may be useful [8].

This study was the first to evaluate the healthcare usage of POTS patients in the ED. We found that patients presented to the ED due to palpitations, syncope, and dyspnea, with some patients presenting to the ED multiple times in two years. Future investigation into ED visits of POTS patients would elucidate whether these visits are appropriate or whether greater patient education could obviate frequent ED visits.

Overall, PCPs felt moderately comfortable managing patients with POTS, but nearly every provider found POTS patients challenging to manage. Overall, PCPs had an accurate picture of the most bothersome POTS symptom. Perhaps to continue to do clinical work, ongoing visits with emphasis on fatigue, body pain, lightheadedness, and less on palpitations are needed. PCPs were also slightly more comfortable managing POTS than POTS patients thought that their PCPs would be, suggesting a disconnect between the comfort level of PCPs and their patients' perception of their competence. Many physicians were unfamiliar with daily behavioral measures and cardiac rehab for the treatment of POTS, and education on the utility of these measures could be a useful future goal for the training of PCPs to provide care to POTS patients.

This study has several limitations. This study included patients of a small group of providers within the Cleveland Clinic neurology department, which increased the proportion of patients who are managed by neurologists compared to other studies. Furthermore, we limited the survey length to just eight questions to increase the likelihood that PCPs would respond to an emailed survey invitation. Due to our desire to shorten the survey, we did not evaluate the objective knowledge or competence of PCPs, but rather their self-perceived competence. We also cannot rule out that PCPs who were more familiar with POTS chose to complete the survey, skewing the results toward more confident providers.

## Conclusions

Overall, this study describes the most common symptoms and the symptoms that have the most significant impact on the quality of life for POTS patients. This study also describes PCPs' thoughts on POTS patients' symptoms that have a negative impact on their quality of life. This study indicates that PCPs are moderately comfortable managing POTS and that there are self-identified gaps in their clinical competency, such as treatment with daily behavioral measures and familiarity with cardiac rehabilitation. Furthermore, recognition among PCPs that fatigue, along with other predominant symptoms of POTS patients, could promote a patient-centered visit, strengthen the PCP-patient partnership, and guide further treatment.

## Figures and Tables

**Figure 4:**
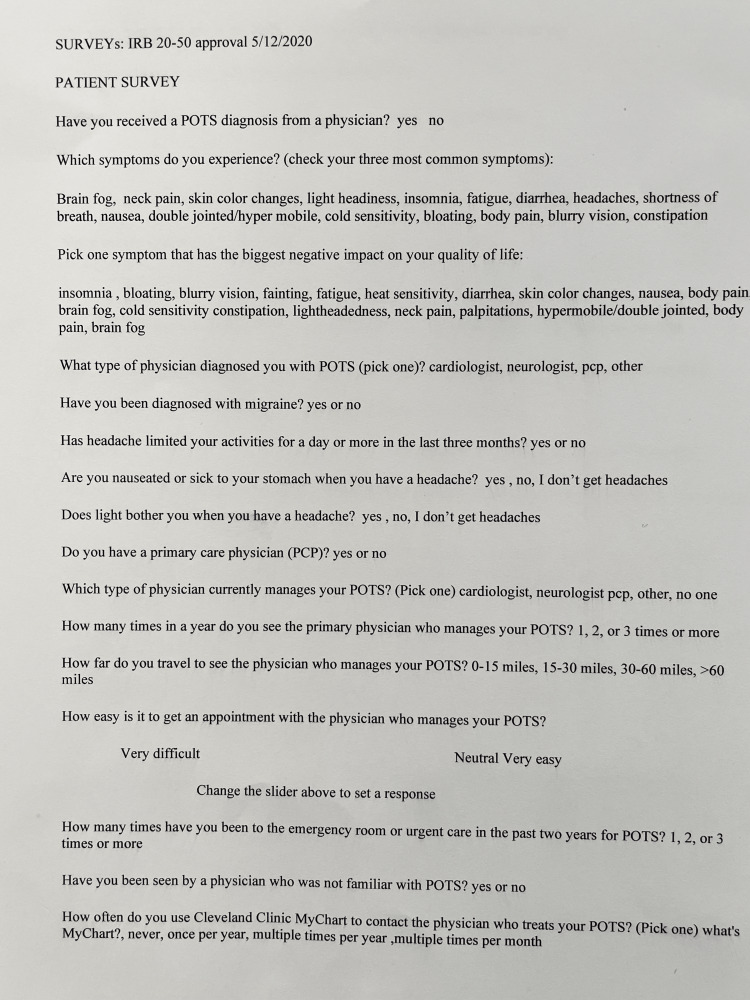
POTS patients' survey. POTS: Postural orthostatic tachycardia syndrome.

**Figure 5:**
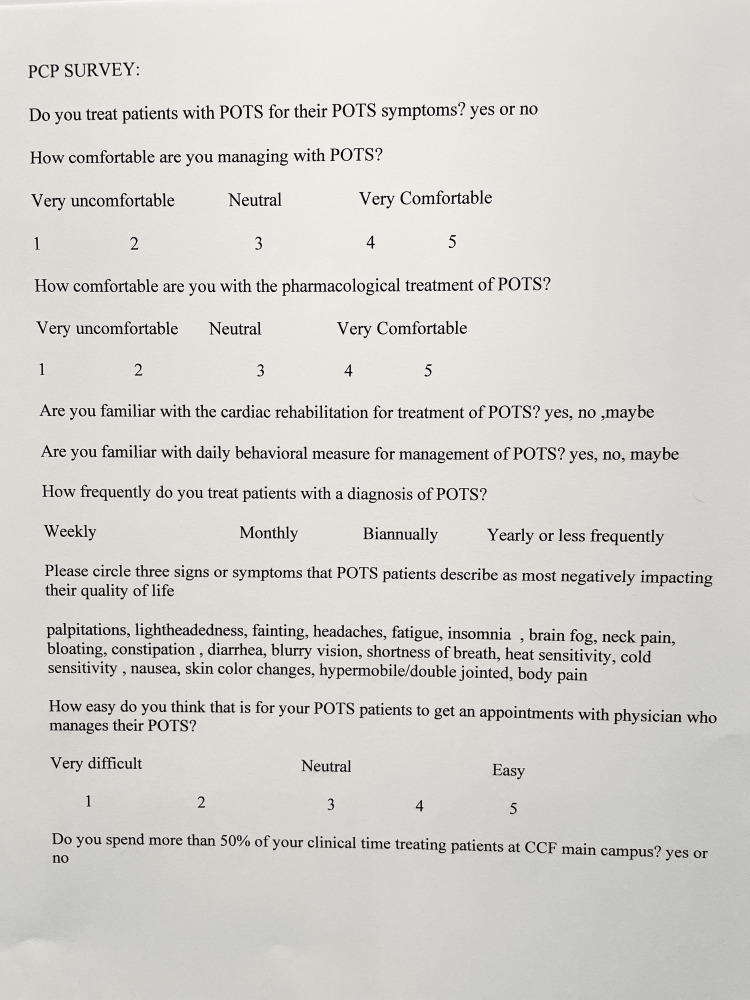
PCP survey. PCP: Primary care physican.

## Appendices

 Appendix 1

Appendix 2
